# The Lung Is Not a Primary Site of Platelet Biogenesis

**DOI:** 10.33549/physiolres.935477

**Published:** 2025-04-01

**Authors:** Luying YE, Houping WANG, Huaan DU, Jianrong HE, Yunxing CAO, Yupei CHEN, Bin SU, He HUANG, Bing CHEN

**Affiliations:** 1 Department of Anesthesia, The Second Affiliated Hospital of Chongqing Medical University, Chongqing, China; 2 Department of Anesthesia, The Third Affiliated Hospital of Chongqing Medical University, Chongqing, China; 3 Department of Cardiology, The Second Affiliated Hospital of Chongqing Medical University, Chongqing, China; 4 Department of Critical Care, The Second Affiliated Hospital of Chongqing Medical University, Chongqing, China

**Keywords:** Platelet, Biogenesis, Megakaryocyte, Lung

## Abstract

Whether the lung is a primary site of platelet (PLT) production is still disputed. To address this question, PLT parameters in blood before and after pulmonary circulation in humans, rats, and rabbits were assessed by automatic hematology analyzers; bone marrow and pulmonary megakaryocytes in humans, mice, rats, and rabbits were evaluated by immunohistochemical staining; and pulmonary megakaryocytes in humans were analyzed by single-cell RNA sequencing. We found that the mean number of PLTs in rats was nearly threefold greater than that in rabbits and humans. The PLT distribution width after pulmonary circulation in humans, rats, and rabbits was consistently less than that before pulmonary circulation. However, except for the PLT population in the left atrium of rats was significantly greater than that in the right ventricle (n=20), the PLT populations between the left and right atria of rats (n=19), rabbits (n=19), and humans (n=24), between the left atrium and right ventricle of rabbits (n=19), and between the inferior vena cava and radial artery of humans (n=93) had no differences. Moreover, megakaryocytes in the lungs of mice, rats, rabbits, and humans were mononuclear, were mainly located perivascularly, and accounted for approximately 3–5 ‰. Their numbers were significantly lower, and their sizes were smaller than those of bone marrow. Conclusively, the lung can produce PLTs, but it is not a primary site of PLT biogenesis. The capability of pulmonary PLT generation differs among species; at least in rats, it is greater than that in rabbits and humans.

## Introduction

Platelets (PLTs) are created from the fragmentations of cytoplasm from mature megakaryocytes [1]. In addition to hemostasis, PLTs also participate in endothelial repair, protect the vascular endothelium, and prevent atherosclerosis, have mechanistic roles in immune responses to disease processes, such as heart transplant rejection, myocardial infarction, aortic aneurysm, peripheral vascular disease, and infections[1–3]. PLTs are essential for maintaining human health; thus, the source of PLTs needs to be clarified.

PLTs are thought to be produced by megakaryocytes in the hematopoietic tissue of the bone marrow[4]. Recently, Lefrançai *et al*. [5] reported that the mouse lung is a primary site for PLT production and accounts for approximately 50 % of total PLT production by using two-photon microscopy *in vivo* and using PLT factor 4 (PF4)-mTmG reporter mice. Moreover, some studies have shown that the number of megakaryocytes in the blood of rats and humans after pulmonary circulation decreases, while the number of PLTs in the blood of cats, rats, and humans after pulmonary circulation increases by counting manually [6–9]. These results indicate that the lung has a robust ability to produce PLTs. In addition, PLT decreases in patients with lung infection [10] and acute respiratory distress syndrome (ARDS) [11] and increases in patients with lung cancer [12–16], which indicates that PLT production is affected by pulmonary diseases.

However, two studies [17,18] using automated blood analyzers revealed no difference in the number of human PLTs before and after pulmonary circulation, which indicates that the human lung has no function in PLT biogenesis. To verify whether the lung can produce PLT, comparisons of blood before and after pulmonary circulation from humans, rats, and rabbits were performed via automatic blood analysis. Moreover, megakaryocytes in bone and lung tissues of humans, rabbits, rats, and mice were detected *via* immuno-histochemical staining, and human lung tissues were assessed by single-cell RNA sequencing (scRNA-seq) analysis. Our results suggested that the lung can produce PLTs, which most likely originate from larger proplatelets, but the lung is not a primary site of PLT biogenesis.

## Materials and Methods

### Ethics

The study protocol was reviewed and approved by the Ethics Committee of the Second Affiliated Hospital of Chongqing Medical University, approval number [2021–82]. All patients gave their informed consent before the study.

### Blood collection and assessment of PLT parameters in animals

All the animals (male) were purchased from Chongqing Laipeter Biotechnology (Chongqing, China). Rats and rabbits were anesthetized with urethane, intubated, and mechanically ventilated. Their chests were opened. Disposable blood collection needles were inserted into the left atrium and right ventricle of 20 rats, and vacuum tubes containing ethylenediamine tetraacetic acid (EDTA) were connected to the needles. Then, 1 ml of blood was collected in each tube and mixed immediately. Blood was collected from the left and right atria of the other 20 rats and from the left atrium, right ventricle, and right atrium of 20 rabbits via the same method. All specimens were examined by an automatic blood cell analyzer (ProCyte Dx, IDEXX Laboratories Inc, Maine, United States) immediately after collection. After blood collection, animals underwent euthanasia through bleeding, followed by confirmation of death through subsequent decapitation.

### Blood collection and assessment of PLT parameters in humans

The inclusion criteria were patients who underwent elective percutaneous catheter ablation for atrial fibrillation under local anesthesia, patients who required inferior vena cava (IVC) and peripheral artery catheterization under local anesthesia for surgery or treatment, or patients who were expected to undergo lobectomy. The exclusion criteria were patients with atrial septal defects, ventricular septal defects, patent ductus arteriosus, tetralogy of Fallot, or transposition of great vessels.

For patients who underwent elective percutaneous catheter ablation for atrial fibrillation, a catheter was inserted into the right atrium, passed through the atrium septum, and passed through the left atrium during the operation. Twenty milliliters of blood were extracted through the catheter with a syringe when the catheter arrived at the right atrium or left atrium; then, 3 ml of blood was collected through the catheter using another syringe and transferred to a vacuum tube containing EDTA immediately. For patients who underwent IVC and peripheral artery catheterization, 20 ml of blood was extracted through the catheter, and 3 ml of blood was collected through the catheter and transferred to a vacuum tube containing EDTA. All the samples were measured by an automatic hematology analyzer (JN-BC6000, Mindray, Shenzhen, China).

### Sample size

According to the report of Kallinikos-Maniatis [7], the PLT in the human aorta was 43.5 × 10^9^/L greater than that in the pulmonary arterial or right atrium. A significance level of 0.01, a power of 0.90, a loss to follow-up rate of 20 %, and a test (inequality) for paired means of PASS11.0 (NCSS Inc., Kaysville, UT, USA) were used to calculate the sample size. The minimum sample size was 5 patients. According to the report of Pedersen [8], the PLT count in the rat aorta was 27.5 × 10^9^/L greater than that in the IVC; the minimum sample size was 9 patients. In this study, at least 20 patients were included for each species.

### Immunohistochemistry and cell counting

The lungs of 4 patients who underwent lobectomy, 4 mice, 4 rats, and 4 rabbits were collected and fixed in 4 % formaldehyde solution. The femurs of 4 mice, 4 rats, and 4 rabbits were collected and fixed in formaldehyde at 4 °C for 24 h, rinsed twice with phosphate-buffered saline, immersed in EDTA decalcifying solution (Solarbio, Beijing, China) at room temperature for 5 days, and then replaced with a new decalcifying solution for 5 days. Formaldehyde-fixed tissues were embedded in paraffin and cut into 5 μm sections. Sections were deparaffinized, incubated with 3 % H_2_O_2_ for 30 min to block endogenous peroxidase activity, retrieved with Tris-EDTA solution (pH 9.0) in a pressure cooker, blocked with 10 % serum, and then incubated with anti-PF4 (A3694), ITGA2B (A11490), and ITGB3 (A19073) antibodies (ABclonal, Wuhan, China) followed by horseradish peroxidase conjugate secondary antibodies (ZSGB-BIO, Beijing). The positive signals were detected by a diaminobenzidine staining kit (ZSGB-BIO, Beijing) and then counterstained with hematoxylin. Microphotographs were taken with a light microscope. The numbers of PF4, ITGA2B, and ITGB3 positive cells per low power field were quantified by Image-Pro Plus 6.0 (Media Cybernetics, Silver Spring, MD, USA) followed by immunohistochemistry data analysis [19]. In addition, PF4, ITGA2B, and ITGB3 immunohistochemical staining data from human sections were obtained from the Human Protein Atlas (http://www.proteinatlas.org).

### Analysis of a molecular cell atlas of the human lungs

Travaglini *et al*. [20] described a human lung molecular cell atlas using 10X Chromium (10X)- or SmartSeq2 (SS2)-based scRNA-seq. 10X scRNA-seq was applied to 65,662 cells, and SS2 scRNA-seq was applied to 9,049 human cells across all lung tissue compartments and circulating blood. The data can be explored in a browser using cellxgene at https://hlca.ds.czbiohub.org/. The positive cells were labeled blue, and the negative cells were labeled green when we input the genes of interest, PF4, ITGA2B, and ITGB3, into the browser. Then, the positive cells were counted manually.

### Statistical analysis

Statistical analysis was performed using SPSS 23.0 (SPSS Inc., Chicago, USA). A non-parametric test (Wilcoxon signed-rank test) were used to compare the PLT parameters in the blood before and after pulmonary circulation. The Mann-Whitney U test was used to compare the difference in the number of positive cells per low magnification between the bone and lung. Statistical significance was defined as P < 0.05.

## Results

### Comparisons of blood PLT parameters before and after pulmonary circulation

To confirm whether the lung is a site of PLT biogenesis, we compared the differences in PLT indices in the blood of the IVC and peripheral artery. A total of 93 patients without atrial fibrillation were enrolled, including 20 patients with ARDS (oxygenation index < 300), 33 patients with lung cancer, and 40 patients with extrapulmonary diseases. Their basic characteristics are shown in Supplemental Table 1. No significant differences in the PLT and red blood cell (RBC)-corrected PLT (PLT/RBC) between the IVC and peripheral artery (Table 1) were detected. After pulmonary circulation, mean PLT volume (MPV), the plateletcrit (PCT), and PLT-large-cell ratio (P-LCR) were statistically increased, while the PLT distribution width (PDW) was significantly decreased (Table 1).

Studies have reported that PLTs decrease in patients with ARDS [11] and increase in patients with lung cancer [12–16]; thus, subgroup analysis was performed. However, there were no significant differences in any of the PLT parameters between the IVC and peripheral artery in patients with ARDS (Table 1). Except for the MPV and P-LCR, which significantly increased after pulmonary circulation, the PLT, PLT/RBC, PCT, and PDW did not significantly differ among patients with lung cancer (Table 1). Moreover, studies have reported that thrombocytopenia may increase PLT production in the lung [21], and the increased biogenesis of PLTs in the lung contributes to thrombocytosis [5]. We performed a subgroup analysis of PLT numbers < 100 × 10^9^/L (Table 1) and > 300 × 10^9^/L (Table 1) and found that all the PLT parameters were not significantly different between the IVC and peripheral artery.

The blood collected at the IVC and the peripheral artery do not accurately represent the blood before and after pulmonary circulation, so we collected blood at the right and left atria. Twenty-four patients who underwent elective percutaneous catheter ablation for atrial fibrillation were enrolled. Their basic characteristics are shown in Supplemental Table 1. None of the PLT indices were significantly different between the right and left atria (Table 2).

To assess whether the differences between before and after pulmonary circulation in animals differ from those in humans, we collected blood from the right atrium, right ventricle, and left atrium of 20 rabbits and 40 rats.

One rabbit and one rat died after anesthesia. The mean number of PLTs in rabbits (Table 3) was close to that in humans (Tables 1 and 2). Except for the PDW in the left atrium of the rabbits was statistically smaller than those in the right atrium (Table 3), other PLT parameters did not significantly differ between the right and left atria or between the right ventricle and left atrium (Table 3). The mean number of PLTs in rats was nearly threefold greater than that in rabbits and humans (Tables 1–4). All PLT parameters did not differ between the right and left atria of the rats (Table 4), but the PLT, PLT/RBC, and PCT in the left atrium of the rats were significantly greater than those in the right ventricle (Table 4). These results indicate that comparing the right ventricle and the left atrium is more accurate in detecting differences in PLT parameters than comparing the right and left atria, and the rat lungs can produce PLTs.

In addition, except for patients with lung cancer and PLT<100*10^9^/L (Table 1), the mean PLT population after pulmonary circulation was greater than that before pulmonary circulation in humans, rats, and rabbits. Furthermore, the mean PDW after pulmonary circulation in humans, rats, and rabbits was consistently lower than that before pulmonary circulation. A lower PDW means that the PLT size varies less [22], which indicates that the PLT size after pulmonary circulation is more uniform than before, and the final step of PLT production from circulating megakaryocytes or larger proplatelets released from the bone marrow can occur in the lung circulation.

### Megakaryocytes in the lungs and bone marrows

**Fig. 1:**
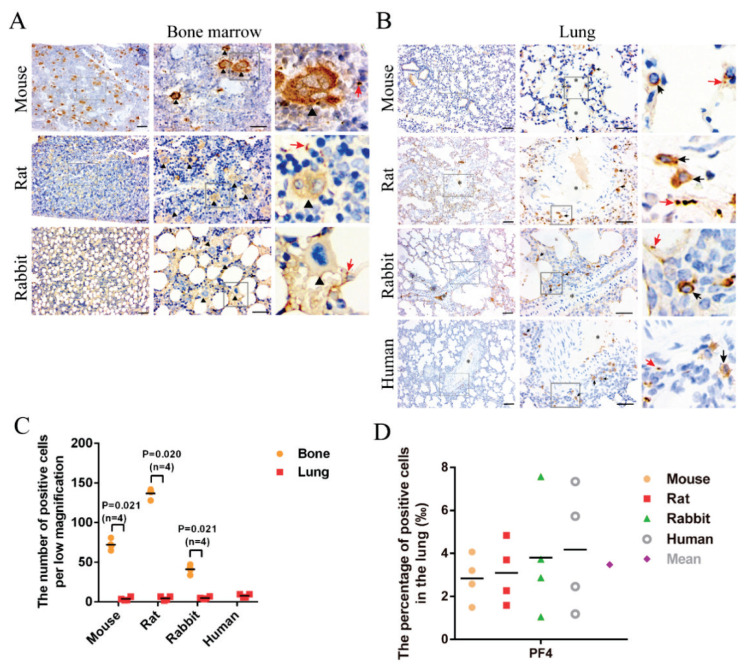
Platelet factor 4 positive (PF4^+^) megakaryocytes in the indicated bone marrows and lungs were detected by immuno-histochemical staining. Sections of bone marrows (**A**) and lungs (**B**) were stained for PF4. The triangles and black arrows indicate PF4^+^ cells. The red arrows indicate platelets or proplatelets. Stars indicate vessels. All the scale bars are 100 μm. (**C**) The numbers of PF4^+^ cells per low magnification in the indicated tissues. (**D**) The percentages and mean number of PF4^+^ cells in the lungs of the indicated species.

To detect whether megakaryocytes were present in the lungs, lung tissues from mice, rats, rabbits, and humans and bone marrows from mice, rats, and rabbits were stained for megakaryocyte markers, including PF4,ITGA2B, and ITGB3 [5,23–25]. Many PF4^+^ (Fig. 1A, triangles), ITGA2B^+^ (Supplemental Fig. 1A, triangles), and ITGB3^+^ (Supplemental Fig. 2A, triangles) cells were found in the bone marrows. All of them had a considerable size (mean diameter of 69 μm) and multinucleated nuclei (mean diameter of 34 μm), which were significantly more prominent than those of the surrounding cells, confirming that PF4, ITGA2B, and ITGB3 are markers of megakaryocytes. However, no PF4^+^ (Fig. 1B), ITGA2B^+^ (Supplemental Fig. 1B), and ITGB3^+^ (Supplemental Fig. 2B) cells with considerable size and multinucleated nucleus as shown in the bone marrows were detected in the lungs of mice, rats, rabbits, and humans. Interestingly, some PF4^+^ (Fig. 1B, black arrows), ITGA2B^+^ (Supplemental Fig. 1B, arrows), and ITGB3^+^ (Supplemental Fig. 2B, arrows) cells with small size (mean diameter of 9 μm) and single nuclei were mainly distributed perivascularly in the lungs of mice, rats, rabbits, and humans, similar to those of other pulmonary cells, which were also shown by Lefrançai *et al*. [5]. The numbers of these cells in the lungs per low-magnification region were significantly lower than that in the bone marrows (Fig. 1C, Supplemental Fig.s 1C and 2C). Moreover, the mean percentages of pulmonary PF4^+^, ITGA2B^+^, and ITGB3^+^ cells accounted for less than 5 ‰ of the total cells (Fig. 1D, Supplemental Figs 1D and 2D).

Similarly, PF4^+^, ITGA2B^+^, and ITGB3^+^ (Supplemental Fig. 3) cells with considerable size and multinucleated nuclei existed in the human bone marrows but did not exist in the human lungs based on the data from the Human Protein Atlas. In addition, these data indicate that PF4, ITGA2B, and ITGB3 antibodies can also identify monocytes (Supplemental Fig. 3, arrows), which is similar to our finding of ITGB3 staining in human lungs (Supplemental Fig. 2B, triangle). Thus, counting pulmonary megakaryocytes needs to be more accurate.

**Fig. 2:**
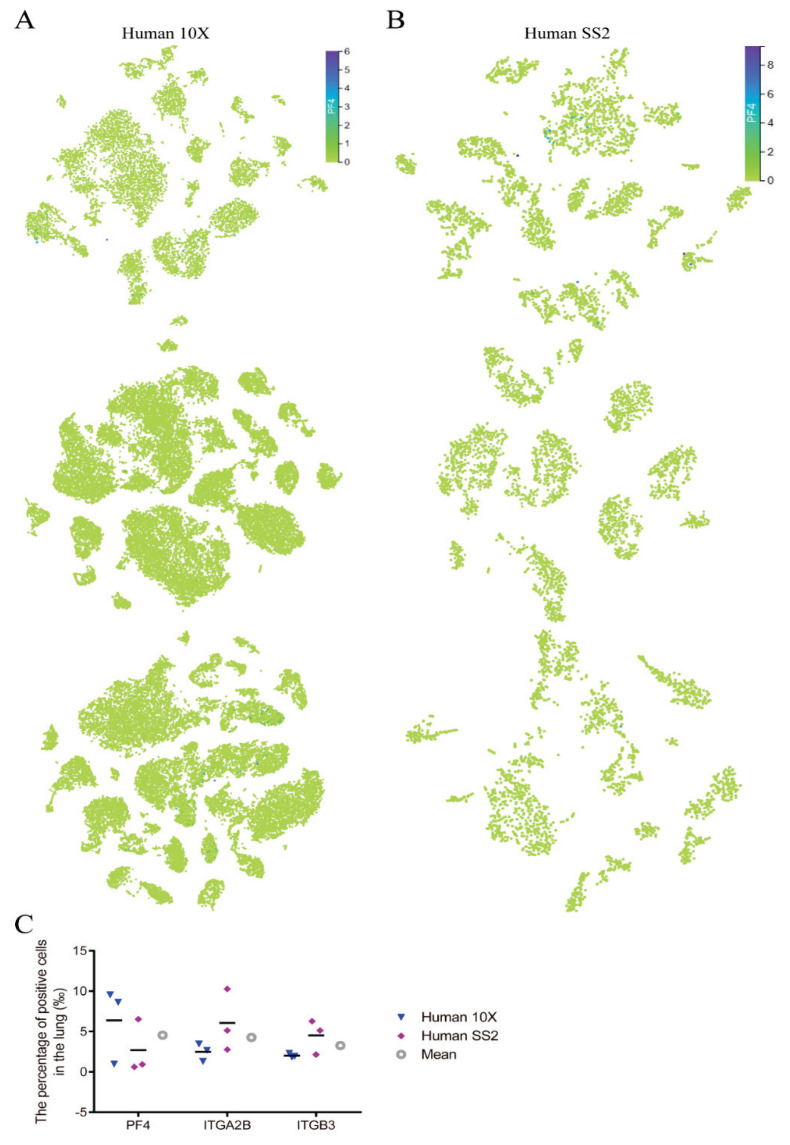
Megakaryocytes in human lungs were detected by single-cell RNA sequencing. Platelet factor 4 (PF4) expression (blue) based on 10X Chromium- (**A**) and SmartSeq2 (SS2)-based (**B**) single-cell RNA sequencing. (**C**) The percentages of PF4^+^, ITGA2B^+^, ITGB3^+^, and mean positive cells based on the indicated methods.

Due to the sizes of pulmonary PF4^+^, ITGA2B^+^, and ITGB3^+^ cells being as small as those of other pulmonary cells, Yeung *et al*. [26] detected fetal and adult megakaryocytes in the lung by scRNA-seq, which demonstrate pulmonary megakaryocytes can be detected by scRNA-seq. To accurately count pulmonary megakaryocytes, we analyzed a published molecular cell atlas of the human lungs using 10X- and SS2-based scRNA-seq to detect all lung tissues and circulating blood. We found that PF4^+^ (Fig.s 2A and 2B), ITGA2B^+^ (Supplemental Fig.s 4A and 4B), and ITGB3^+^ (Supplemental Fig.s 5A and 5B) cells were sparse and occupied approximately 3–5 ‰ of the population (Fig. 2C), which was similar to that observed by immunohistochemical staining. These results demonstrated that the number of pulmonary megakaryocytes was sparse, and their morphology was distinguished entirely from that in the bone marrow.

## Discussion

It is generally believed that if the lung has the ability to produce PLTs, then the blood after pulmonary circulation will have more PLTs than the blood before entering the pulmonary circulation [6–8,17,18]. In this study, the mean PDW after pulmonary circulation in humans, rats, and rabbits was consistently lower than that before pulmonary circulation, which means that the PLT size is more uniform after pulmonary circulation, and the final step of PLT production can occur in the lung circulation [22]. However, except for the blood PLT population in the left atrium of the rats was statistically greater than those in the right ventricle, the blood PLT population did not significantly differ between the IVC and radial artery, the right and left atria of humans, between the right and left atria of rats and rabbits, or between the right ventricle and left atrium of rabbits. Therefore, our data indicate that the lung can produce PLTs but is not a primary site of PLT biogenesis. Moreover, its capability of PLT biogenesis differs among species; at least in rats, it is greater than that in rabbits.

Lefrançais *et al*. [5] reported that the increased biogenesis of PLTs in the lung contributed to thrombocytosis. Thus, the different capabilities of PLT production among species may be attributed to the fact that the number of PLTs in rats was nearly threefold greater than that in rabbits. According to our data, comparing the right ventricle and the left atrium is more accurate in detecting differences in PLT parameters than other comparisons. However, right ventricle blood from humans was unavailable, which is a limitation of our study. Nevertheless, the mean number of PLTs in humans is similar to that in rabbits; thus, the PLT production in humans may be similar to that in rabbits and lower than that in rats.

Our results were different from those of two studies [6,7] in that 80–89 % of patients had more PLTs in the aorta than in the vein or right atrium (from 30 × 10^9^/L to 80 × 10^9^/L), which were counted manually, but were consistent with three studies [17,18,27] in which no differences in PLT counts were detected between the human pulmonary arterial and peripheral arteries or between the right and left atria in children with congenital heart disease by using an automated blood analyzer. Moreover, our results were the same as those of Pedersen’s study [8], in which the PLT populations in the rat aorta were greater than that in the IVC (an average of 27.5 × 10^9^/L). These data demonstrated that the PLT population in rats after pulmonary circulation was significantly greater than that before pulmonary circulation, but there was no difference in the PLT population in humans, which indicates that the PLT production ability in humans is lower than that in rats.

PLT production in the lung circulation may originate from circulating megakaryocytes or larger proplatelets released from the bone marrow. Here, we found that PF4^+^, ITGA2B^+^, and ITGB3^+^ megakaryocytes in the lungs of mice, rats, rabbits, and humans were sparse and occupied approximately 3–5‰ according to immunohistochemical staining and analysis of public human scRNA-seq, which is inconsistent with the difference in pulmonary PLT generation between rats and rabbits or between rats and humans. Moreover, they were mainly distributed perivascularly; the nuclei of these megakaryocytes in the lung were mononuclear and as small as those in the other pulmonary cells (mean diameter of 9 μm), while those in the bone marrows were considerable (mean diameter of 69 μm) and had multinucleated nuclei (mean diameter of 34 μm); similar to the findings of three studies [5,25,26]. According to the study of Dejima *et al*. [24], the PF4^+^, ITGA2B^+^, and ITGB3^+^ megakaryocytes in the lung were immature. Taken together, these data indicate that PF4^+^, ITGA2B^+^, and ITGB3^+^ megakaryocytes in the lung have a limited ability to generate PLTs and that PLT production in the lung circulation mainly originates from larger proplatelets. In addition, except for platelet production, evidence from scRNA-seq indicated that pulmonary megakaryocyte functions for immunoregulatory and stem cell niches [28]. Further studies need to explore their functions in pulmonary diseases.

Moreover, we found that the number of pulmonary megakaryocytes was significantly less than that in the borrow marrow, similar to the findings of Potts *et al*. [25]. They also found that membrane budding of megakaryocytes in the bone marrow supplied most of the PLT biomass, accounting for 89 % of the estimated 3.82 × 10^7^ PLTs per hour required to maintain stable circulating numbers. In addition, a recent study [29] reported that a small fraction of megakaryocytes reside in the lung and contribute minimally to the PLT pool. These data indicate that the lung is not the primary site of PLT biogenesis [29], which is different from the findings of the study of Lefrançai *et al*. [5], in which PLT production in the lung accounted for approximately 50 % of the total PLT production.

In total, megakaryocytes in the lungs are mononuclear, are mainly located perivascularly, and account for approximately 3–5‰ of the total lung cells. Their numbers are significantly lower, and their sizes are smaller than those of bone marrow. The lung can produce PLTs, which most likely originate from larger proplatelets, but the lung is not a primary site of PLT biogenesis based on comparing platelet parameters before and after pulmonary circulation in humans, rats, and rabbits. In addition, the capability of pulmonary PLT generation differs among species; at least in rats, it is greater than that in rabbits and humans.

## Supplementary Information



## Figures and Tables

**Table 1:** Comparisons of platelet parameters between the inferior vena cava and radial artery in patients without atrial fibrillation. IVC: inferior vena cava; PLT: platelet; RBC: red blood cell; PDW: platelet distribution width; MPV: mean platelet volume; PCT: plateletcrit; P-LCR: platelet-large-cell ratio; ARDS: acute respiratory distress syndrome.

	IVC	Radial artery	Radial artery minus IVC	P
**1. Patients without atrial fibrillation (n=93)**				

*PLT (10* *^9^* */L)*	204.67±86.38	205.75±85.19	1.09±11.71	0.560
*PLT/RBC (%)*	5.24±2.87	5.25±2.80	0.01±0.31	0.390
*PDW*	16.32±0.43	16.27±0.37	−0.59±0.25	0.041
*MPV (fl)*	10.96±1.07	10.99±1.16	0.03±0.52	0.014
*PCT (%)*	0.220±0.081	0.222±0.080	0.003±0.012	0.034
*P-LCR (%)*	32.48±7.71	32.80±8.32	0.32±3.67	0.015

**2. Patients with ARDS (n=20)**				

*PLT (10* *^9^* */L)*	189.20±152.65	190.85±149.73	1.65±10.56	0.386
*PLT/RBC (%)*	6.47±5.36	6.47±5.18	−0.001±0.394	0.422
*PDW*	16.64±0.49	16.54±0.45	−0.10±0.30	0.218
*MPV (fl)*	10.77±1.12	10.86±1.20	0.10±0.60	0.169
*PCT (%)*	0.194±0.137	0.198±0.135	0.004±0.015	0.197
*P-LCR (%)*	31.55±7.61	32.44±8.19	0.89±3.84	0.218

**3. Patients with lung cancer (n=33)**				

*PLT (10* *^9^* */L)*	208.70±49.48	206.88±50.96	−1.82±7.95	0.328
*PLT/RBC (%)*	4.87±1.21	4.83±1.27	−0.04±0.20	0.421
*PDW*	16.19±0.32	16.17±0.31	−0.02±0.17	0.224
*MPV (fl)*	11.01±1.10	11.18±1.13	0.17±0.25	0.000
*PCT (%)*	0.228±0.0539	0.229±0.0529	0.001±0.010	0.505
*P-LCR (%)*	32.51±7.83	33.87±8.12	1.36±0.20	0.000

4. Patients with PLT<100*10 **^9^** **/L (n=6)**				

*PLT (10* *^9^* */L)*	57.83±18.61	57.50±23.06	−0.33±7.12	0.893
*PLT/RBC (%)*	1.78±0.55	1.76±0.66	−0.02±0.24	0.833
*PDW*	17.15±0.64	16.80±0.43	−0.35±0.39	0.072
*MPV (fl)*	11.28±0.64	10.72±1.27	−0.57±0.96	0.115
*PCT (%)*	0.066±0.024	0.063±0.029	−0.003±0.009	0.500
*P-LCR (%)*	34.43±4.60	32.17±7.17	−2.27±5.65	0.463

5. Patients with PLT>300*10 **^9^** **/L (n=9)**				

*PLT (10* *^9^* */L)*	383.22±120.00	383.44±112.30	0.22±12.48	0.953
*PLT/RBC (%)*	11.73±5.01	11.63±4.71	−0.10±0.49	0.953
*PDW*	15.98±0.32	15.91±0.30	−0.07±0.18	0.293
*MPV (fl)*	9.77±0.80	9.83±0.70	0.07±0.17	0.221
*PCT (%)*	0.370±0.102	0.373±0.092	0.002±0.162	0.635
*P-LCR (%)*	24.47±5.18	24.60±4.45	0.13±1.21	0.859

**Table 2:** Comparisons of platelet parameters between the right atrium and left atrium in patients with atrial fibrillation (n=24). PLT: platelet; RBC: red blood cell; PDW: platelet distribution width; MPV: mean platelet volume; PCT: plateletcrit; P-LCR: platelet-large-cell ratio.

	Right atrium	Left atrium	Left atrium minus Right atrium	P
*PLT (10* *^9^* */L)*	176.00±49.78	177.54±53.48	1.54±11.09	0.440
*PLT/RBC (%)*	4.27±1.22	4.29±1.22	−1.03±3.40	0.637
*PDW*	16.20±0.35	16.19±0.36	−0.01±0.20	0.887
*MPV (fl)*	11.32±1.61	11.18±1.50	−0.14±0.45	0.161
*PCT (%)*	0.195±0.424	0.193±0.427	−0.002±0.012	0.758
*P-LCR (%)*	34.46±10.91	33.43±10.37	−1.03±3.40	0.219

**Table 3:** Comparisons of platelet parameters before and after pulmonary circulation in rabbits (n=19). PLT: platelet; RBC: red blood cell; PDW: platelet distribution width; MPV: mean platelet volume; PCT: plateletcrit; P-LCR: platelet-large-cell ratio.

1. Between the right atrium and left atrium				
	Right atrium	Left atrium	Left atrium minus Right atrium	P
*PLT (10* *^9^* */L)*	359.21±95.54	360.37±96.88	1.15±54.46	0.888
*PLT/RBC (%)*	7.86±2.70	7.85±2.73	−1.36±100.22	0.809
*PDW*	7.39±3.20	6.92±1.82	−0.47±1.53	0.035
*MPV (fl)*	8.17±0.59	8.19±0.62	0.02±0.18	0.753
*PCT (%)*	0.285±0.079	0.294±0.085	0.009±0.029	0.135
*P-LCR (%)*	8.46±8.52	7.78±5.75	−0.68±3.58	0.601

**2. Between the right ventricle and left atrium**				
	**Right ventricle**	**Left atrium**	**Left atrium minus Right ventricle**	**P**

*PLT (10* *^9^* */L)*	355.95±116.63	360.37±96.88	4.42±56.53	0.758
*PLT/RBC (%)*	7.66±2.94	7.85±2.73	18.97±113.68	0.586
*PDW*	7.31±3.04	6.92±1.82	−0.39±1.41	0.185
*MPV (fl)*	8.19±0.65	8.19±0.62	−0.01±0.20	1.000
*PCT (%)*	0.298±0.098	0.294±0.085	0.005±0.028	0.415
*P-LCR (%)*	8.66±8.46	7.78±5.75	−0.88±3.41	0.432

**Table 4:** Comparisons of platelet parameters before and after pulmonary circulation in rats (n=19). PLT: platelet; RBC: red blood cell; PDW: platelet distribution width; MPV: mean platelet volume; PCT: plateletcrit; P-LCR: platelet-large-cell ratio.

1. Between the right atrium and left atrium				
	Right atrium	Left atrium	Left atrium minus Right atrium	P
PLT (*10 *^9^* */L)*	1012.16±110.53	1013.79±143.88	1.63±112.59	0.984
*PLT/RBC (%)*	11.41±1.63	11.40±1.78	−0.01±1.27	0.936
*PDW*	8.05±0.61	7.99±0.47	−0.06±0.38	0.713
*MPV (fl)*	8.49±0.35	8.44±0.35	−0.05±0.21	0.172
*PCT (%)*	0.857±0.078	0.855±0.117	−0.002±0.094	0.840
*P-LCR (%)*	7.53±1.52	7.31±1.65	−0.25±1.40	0.878

**2. Between the right ventricle and left atrium**				
	**Right ventricle**	**Left atrium**	**Left atrium minus Right ventricle**	**P**

PLT (*10 *^9^* */L)*	895.75±138.95	924.9±141.88	29.15±51.67	0.025
*PLT/RBC (%)*	9.38±2.21	9.66±2.13	0.29±0.51	0.037
*PDW*	8.21±0.76	8.15±0.63	−0.06±0.37	0.136
*MPV (fl)*	9.03±0.46	9.00±0.40	−0.03±0.22	0.548
*PCT (%)*	0.809±0.120	0.833±0.130	0.024±0.047	0.043
*P-LCR (%)*	7.83±2.41	7.67±2.23	−0.16±1.73	0.709
