# Supplementary material for: The Lung Is Not a Primary Site of Platelet Biogenesis
Source: Physiol Res. 2025 Apr 1;74(2):263–73. doi: 10.33549/physiolres.935477 (PMC12148122; doi:10.33549/physiolres.935477)
Supplement: Supplementary file 1 [file PR74_263_Supplemental_data.pdf]

Supplemental Table 1. Baseline characteristics of patients. ARDS: acute respiratory distress syndrome; BMI: body max index; OI: oxygenation. Index; IVC: inferior vena cava.

	**Patients with atrial fibrillation (n=24)**	**Patients without atrial fibrillation (n=93)**	**Patients with ARDS (n=20)**	**Patients with lung cancer (n=33)**	**Patients without lung cancer or ARDS (n=40)**
Age (year)	63.75±11.79	57.71±13.51	63.00±13.55	56.94±11.77	54.93±14.15
Sex (male/female)	15/9 (62.50/37.50)	54/39 (58.06/41.94)	11/9 (55.00/45.00)	20/13 (60.61/39.39)	23/17 (57.50/42.50)
Height (cm)	164.46±7.56	162.71±8.34	163.60±8.81	162.33±8.87	162.32±7.77
Weight (Kg)	66.16±10.52	64.64±12.02	63.75±14.69	65.24±11.03	64.32±11.47
BMI (Kg/m^2)	24.41±3.22	24.26±3.05	23.54±3.57	24.60±2.44	24.32±3.18
OI			181.20±55.42		
Blood collection site	left atrium and right atrium	radial artery and IVC	radial artery and IVC	radial artery and IVC	radial artery and IVC

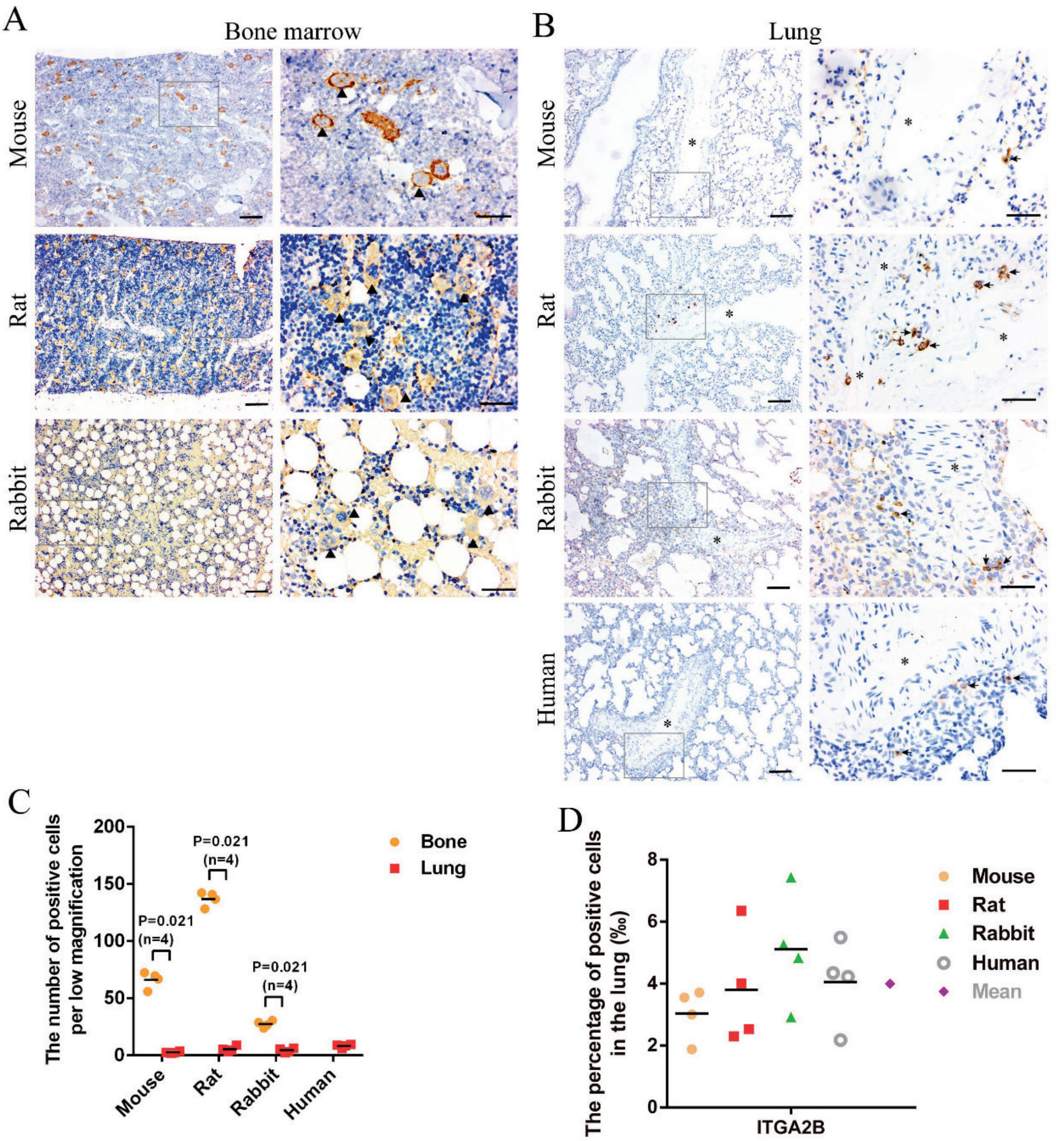

Supplemental Fig. 1. ITGA2B$^+$ megakaryocytes in the indicated bone marrows and lungs were detected by immunohistochemical staining. Sections of bone marrows (A) and lungs (B) were stained for ITGA2B. The triangles and arrows indicate ITGA2B$^+$ cells. Stars indicate vessels. All the scale bars are 100 μm. (C) The numbers of ITGA2B$^+$ cells per low magnification in the indicated tissues. (D) The percentages and mean number of ITGA2B$^+$ cells in the lungs of the indicated species.

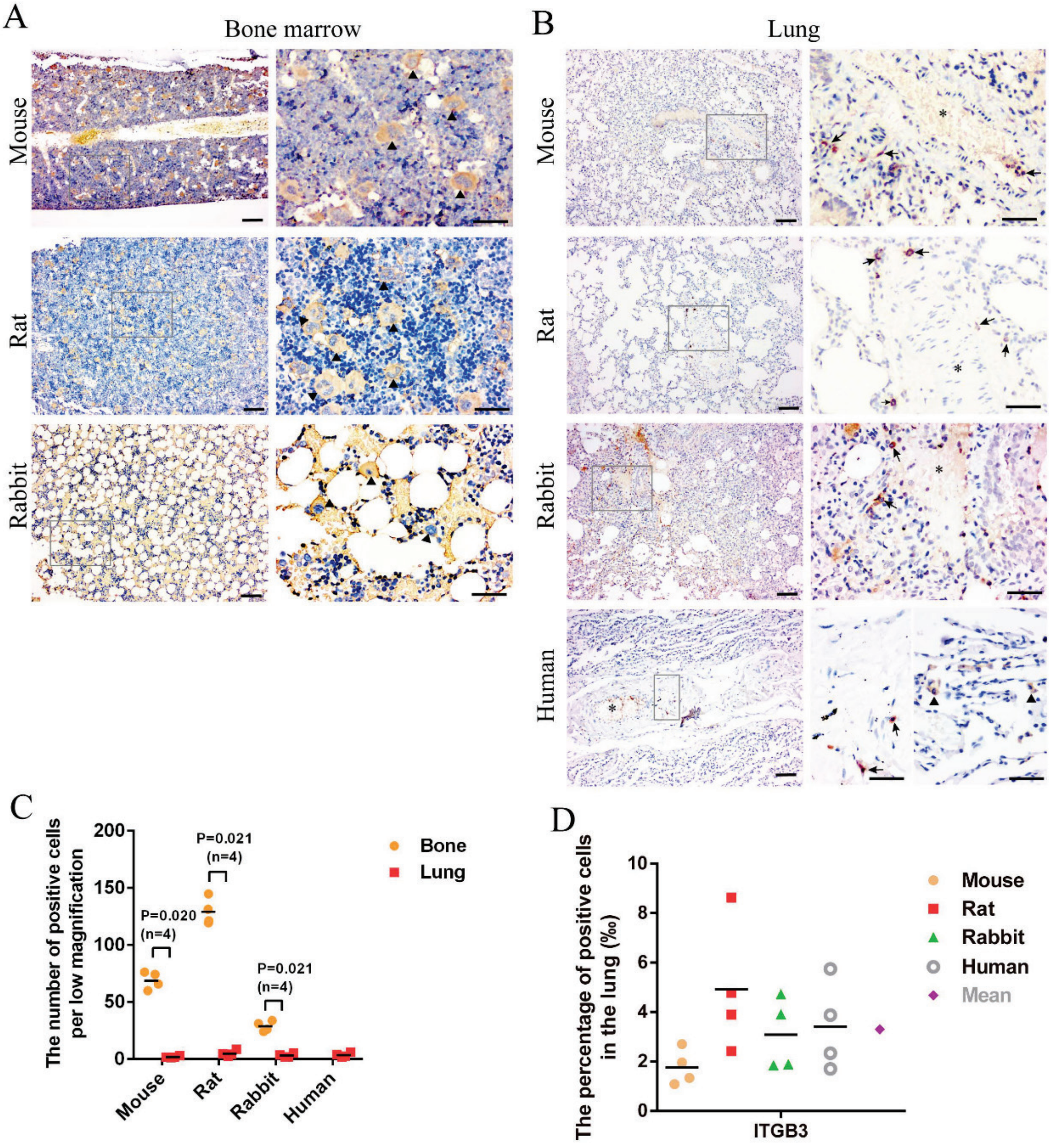

Supplemental Fig. 2. ITGB3$^+$ megakaryocytes in the indicated bone marrows and lungs were detected by immunohistochemical staining. Sections of bone marrows (A) and lungs (B) were stained for ITGB3. Triangles and arrows indicate ITGB3$^+$ cells. Stars indicate vessels. All the scale bars are 100 μm. (B) The numbers of ITGB3$^+$ cells per low magnification in the indicated tissues. (C) The percentages and mean number of ITGB3$^+$ cells in the lungs of the indicated species.

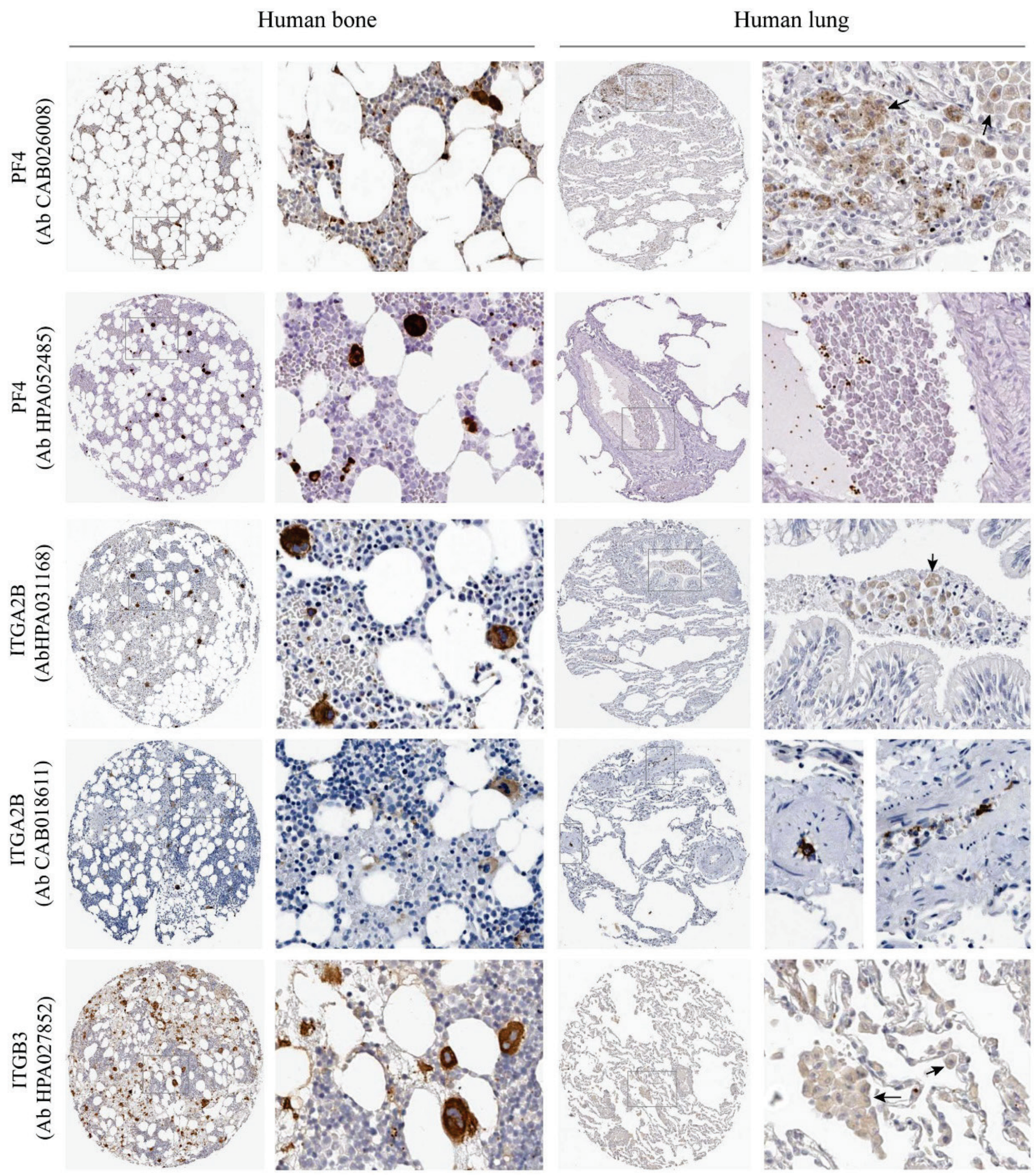

Supplemental Fig. 3. PF4⁺, ITGA2B⁺, and ITGB3⁺ megakaryocytes in human bone marrows and lungs detected by immunohistochemical staining were downloaded from the Human Protein Atlas (http://www.proteinatlas.org). Arrows indicate positive cells.

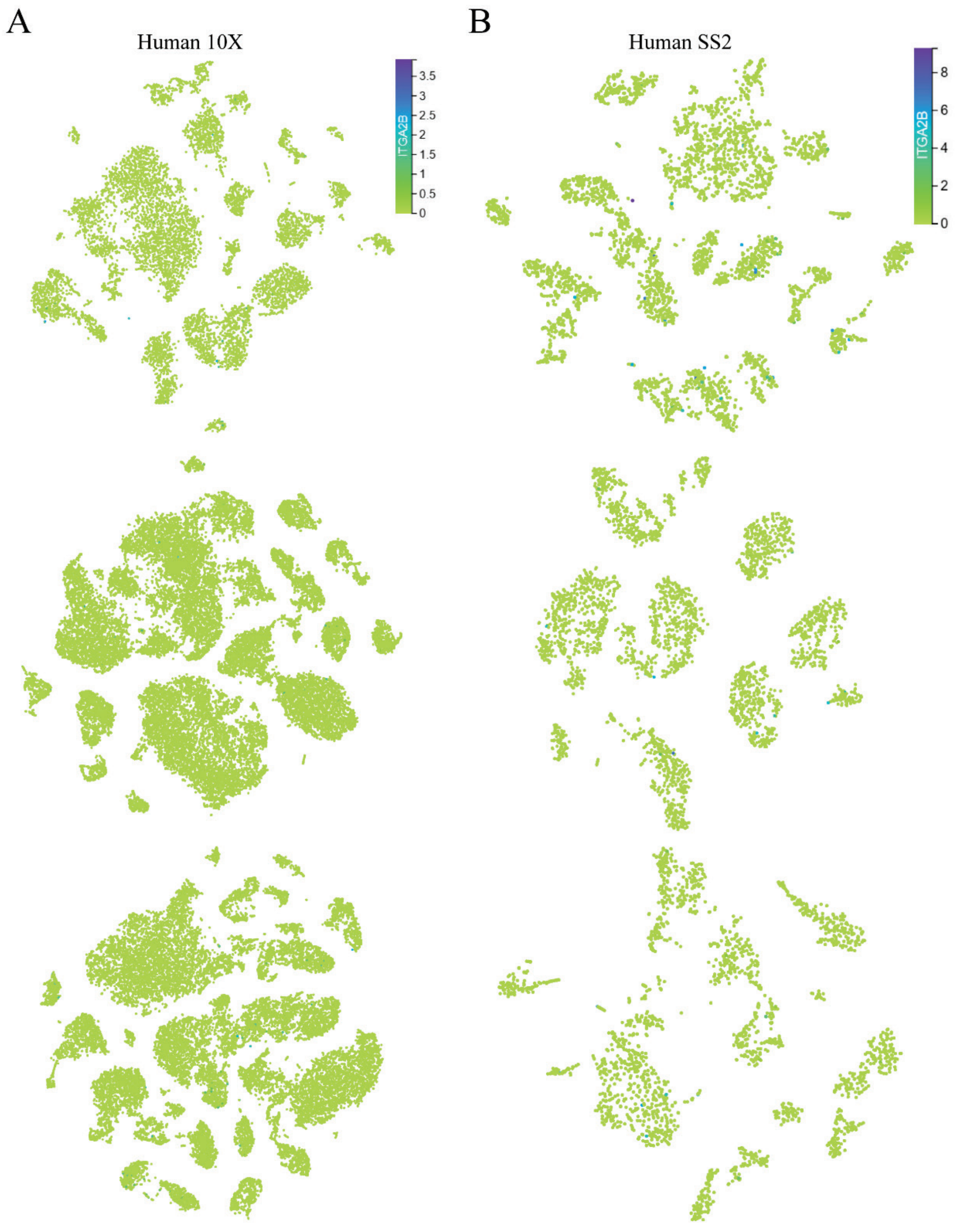

Supplemental Fig. 4. ITGA2B^{+} megakaryocytes in human lungs detected by single-cell RNA sequencing. ITGA2B expression (blue) based on 10X Chromium- (A) and SmartSeq2 (SS2)-based (B) single-cell RNA sequencing.

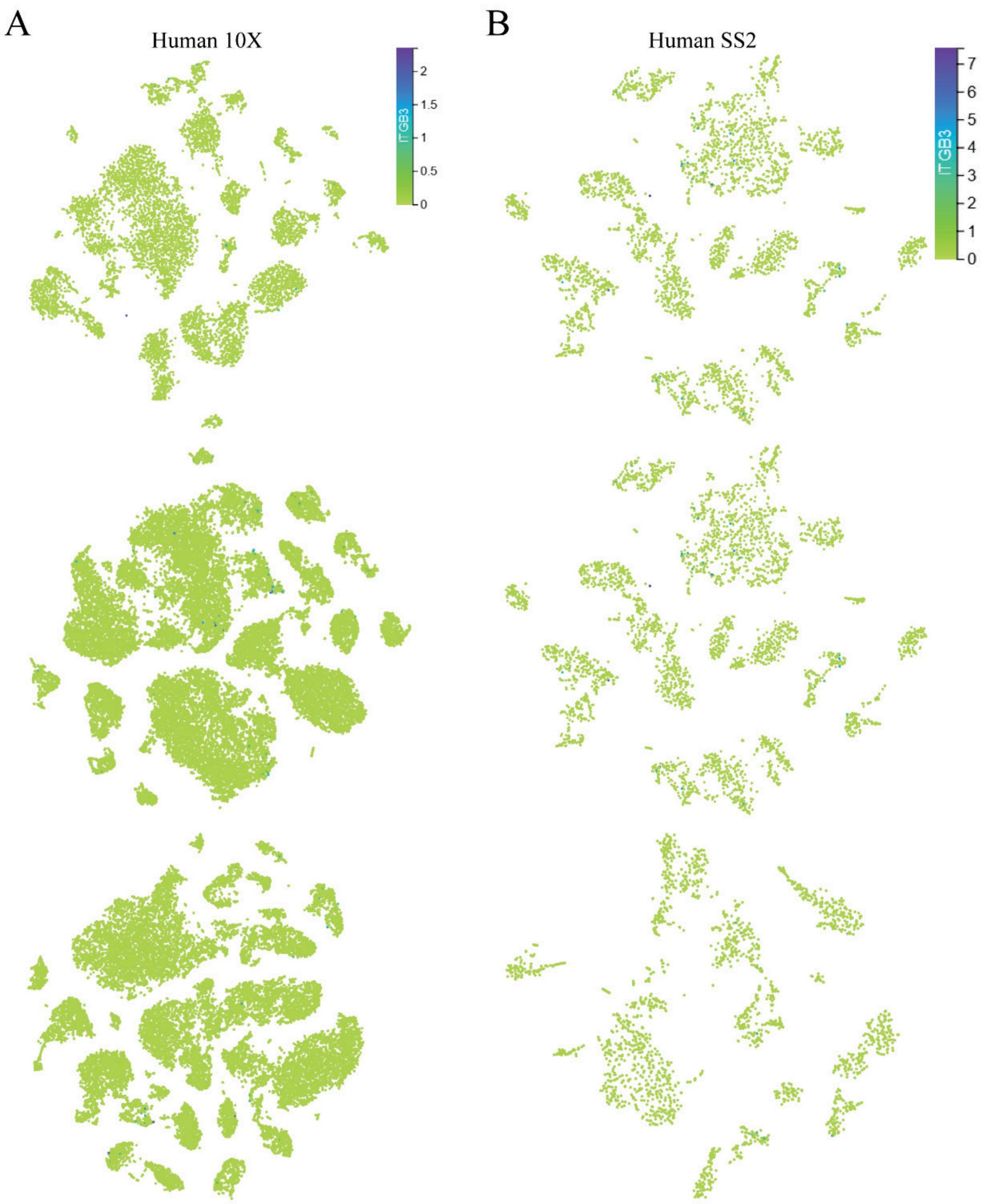

Supplemental Fig. 5. ITGB3$^+$ megakaryocytes in human lungs detected by single-cell RNA sequencing. ITGB3 expression (blue) based on 10X Chromium- (A) and SmartSeq2 (SS2)-based (B) single-cell RNA sequencing.
